# Surgery plus anesthesia induces loss of attention in mice

**DOI:** 10.3389/fncel.2015.00346

**Published:** 2015-09-08

**Authors:** Quan Ren, Mian Peng, Yuanlin Dong, Yiying Zhang, Ming Chen, Ning Yin, Edward R. Marcantonio, Zhongcong Xie

**Affiliations:** ^1^Department of Anesthesia, Zhongda Hospital, Southeast UniversityNanjing, China; ^2^Geriatric Anesthesia Research Unit, Department of Anesthesia, Critical Care and Pain Medicine, Massachusetts General Hospital and Harvard Medical SchoolCharlestown, MA, USA; ^3^Department of Anesthesia, Zhongnan Hospital of Wuhan UniversityWuhan, China; ^4^Urology Department, Zhongda Hospital, Southeast UniversityNanjing, China; ^5^Divisions of General Medicine and Primary Care and Gerontology, Department of Medicine, Beth Israel Deaconess Medical Center and Harvard Medical SchoolBoston, MA, USA

**Keywords:** anesthesia, surgery, attention, synuclein, S100β

## Abstract

There is a need to develop animal models to study postoperative delirium. Inattention is one of the symptoms of delirium. Increases in the levels of α-synuclein and S100β have been reported to be associated with delirium. Therefore, we set out to determine the effects of surgery plus general anesthesia on the behavioral changes (including loss of attention) in mice and on the levels of α-synuclein and S100β in the brain tissues of these mice. C57BL/6J mice (2- to 8-months-old) had a simple laparotomy plus isoflurane anesthesia. The behavioral changes, including attention level and the speed of movements, were determined 12, 24, and 48 h after the surgery plus anesthesia in the mice. The levels of α-synuclein and S100β in the cortex of these mice following the surgery plus anesthesia were determined by Western blot analysis. We found that there was a loss of attention at 24, but not 12 or 48 h following the surgery plus anesthesia (49% ± 5 vs. 33% ± 2.9, *P* = 0.011, *N* = 12) in the mice without significantly affecting the speed of their movements. There were increases in the levels of total α-synuclein (139% ± 33.5 vs. 100% ± 13.7, *P* = 0.037, *N* = 6) and S100β (142% ± 7.7 vs. 100% ± 6, *P* = 0.002, *N* = 6) in the cortex of the mice 12 h following the surgery plus anesthesia. These findings suggested that the surgery plus isoflurane anesthesia might induce behavioral and biochemical/cellular changes associated with delirium. We could use the surgery plus anesthesia in mice to develop an animal model to study postoperative delirium.

## Introduction

Postoperative delirium, a common postoperative complication (Marcantonio et al., 1994; Liu and Leung, 2000), has independent and adverse effects on short- and long-term mortality and morbidity, including poor functional recovery, postoperative cognitive dysfunction, deterioration in quality of life, and increased costs of medical care (Inouye, 2006; Ansaloni et al., 2010; Jankowski et al., 2011; Saczynski et al., 2012, reviewed in Deiner and Silverstein, 2009; Vasilevskis et al., 2012). However, the neuropathogenesis and targeted intervention(s) for postoperative delirium remain largely to be determined, partially owing to the lack of animal models to study postoperative delirium. The establishment of animal model(s) to study postoperative delirium, therefore, would likely facilitate investigations into the neuropathogenesis of postoperative delirium, and consequently, could lead to the development of targeted intervention(s).

Thus far, few animal models related to the study of delirium have been established. Murray et al. reported that the “paddling” T-maze alternation task might be used to detect acute working memory deficits induced by systemic administration of bacterial endotoxin lipopolysaccharide (LPS), which might be utilized to study delirium (Murray et al., 2012). The attentional set-shifting task (AST), another potential animal model that could be used to study delirium (Culley et al., 2014), includes tests of discrimination problems, which are based on stimulus dimensions (e.g., odor and intra-and extra-dimensional cues). It has been suggested that AST is similar to the Wisconsin Card Sorting Test (WCST) (Culley et al., 2014), a test used clinically to detect impairments in attention and executive function due to prefrontal cortex damage or dysfunction in patients (Bourne and Milner, 1963; Roberts et al., 1988).

The Confusion Assessment Method (CAM) algorithm, developed in 1990 (Inouye et al., 1990) and widely utilized to determine the presence of delirium in human patients, consists of four clinical criteria: (1) acute onset and fluctuating course, (2) inattention, (3) disorganized thinking, and (4) altered level of consciousness. For delirium to be defined by this algorithm, both the first and the second (inattention) criteria have to be present, plus either the third and/or the fourth criterion. We therefore set out to determine the effects of the surgery (simple laparotomy) under isoflurane anesthesia on attention level in mice in the present experiment. Clinical investigations have shown that elevations in the levels of α-synuclein (Sunwoo et al., 2013) and S100β (Hall et al., 2013; Khan et al., 2013) could be associated with postoperative delirium. Thus, we also assessed whether the surgery plus anesthesia would increase the levels of α-synuclein and S100β in the brain tissues of mice.

The main objective of the current pilot studies was to determine whether surgery plus anesthesia was able to induce behavioral (e.g., attention) and biochemical/cellular (α-synuclein and S100β) changes associated with delirium. The long-term goal of our studies was to establish an animal model of perioperative factors, e.g., anesthesia, surgery, pain and sleep deprivation, which could recapitulate, at least partially, the symptoms of postoperative delirium observed in humans. The primary hypothesis was that the surgery plus anesthesia in mice was able to induce behavioral changes (e.g., loss of attention) and biochemical/cellular changes (e.g., increase in the levels of α-synuclein and S100β) in the mice.

## Materials and methods

### Mice

The animal protocol was approved by the Standing Committee on Animals at Massachusetts General Hospital, Boston, Massachusetts. C57BL/6J mice (2-to 8-months-old, female, The Jackson Laboratory, Bar Harbor, ME) were randomly assigned to the surgery plus anesthesia group or the sham group. The mice were housed in a controlled environment (20–22°C; 12 h of light/dark on a reversed light cycle) for 1 week prior to the studies. The maintenance and handling of the mice were consistent with the National Institute of Health guidelines, and all efforts were made to minimize the number of animals needed in the studies. Power analyses used to establish experimental group sizes are described below in the Statistics section.

### Mice surgery plus anesthesia

We performed a simple laparotomy under isoflurane anesthesia using the methods described in our previous studies, (Xu et al., 2014a,b) with modifications. Specifically, anesthesia was induced and maintained with 1.4% isoflurane in 100% oxygen in a transparent acrylic chamber. Fifteen minutes after the induction, the mouse was moved out of the chamber, and isoflurane anesthesia was maintained via a cone device. One 16-gauge needle was inserted into the cone near the nose of the mouse to monitor the concentration of isoflurane. A longitudinal midline incision was made from the xiphoid to the 0.5 cm proximal pubic symphysis on the skin, abdominal muscles and peritoneum. Then, the incision was sutured layer by layer with 5-0 Vicryl thread. At the end of the procedure, EMLA cream (2.5% lidocaine and 2.5% prilocaine) was applied to the incision wound every 8 h for 2 days to treat the pain associated with the incision. Our previous studies found that neither this type of surgery (Xu et al., 2014a,b) nor anesthesia with 1.4% isoflurane (Xie et al., 2008) significantly disturbs the blood pressure or the blood gas values of the mice. The surgery for each mouse lasted about 10 min. After the surgery plus isoflurane anesthesia, the mouse was put back into the anesthesia chamber to receive the rest of the anesthesia consisting of 1.4% isoflurane in 100% oxygen for up to 2 h. The temperature of the anesthetizing chamber was controlled (DC Temperature Control System; FHC, Bowdoinham, Maine) in order to maintain the rectal temperature of the mice at 37 ± 0.5°C during the anesthesia. After recovering from the anesthesia, each mouse was returned to a home cage with food and water available *ad libitum*. The mice in the sham group were placed in a similar transparent acrylic chamber with 100% O_2_ supply for 2 h.

### Behavioral test

The tests used to assess the attention levels of the mice were performed as described by Millecamps et al. (2004). Specifically, the studies were performed in an open field chamber (40 × 40 × 40 cm), with four objects placed at trigonal points on the two diagonal lines of the chamber floor. A video camera, which was linked to the Any-Maze animal tracking system software (Stoelting Co., Wood Dale, IL), was installed 60 cm above the chamber to monitor and analyze the movement of each of the mice and the time spent with each object. Each mouse was put into the chamber with four identical objects for 10 min per day on three consecutive days for familiarization before the surgery plus isoflurane anesthesia, which was performed on the third day after the third familiarization. The new object exploration time was measured 12, 24, and 48 h after the surgery plus anesthesia. For the attention test, one of the familiar objects was replaced by a new object with the same size as the familiar objects, but with a different shape, color, and texture (the novel object). During the test, each of the mice was introduced to the chamber for 5 min. The time the mouse spent with each of the four objects was recorded and analyzed. The attention level of each mouse was defined as the percentage of time the mouse spent exploring the new object (new object exploration time) in comparison with the total time of exploration of the four objects (total object exploration time).

### Open field tests

Open field tests were performed after each of the attention tests with the mice (e.g., 12, 24, and 48 h after the surgery plus anesthesia). The experiments were conducted in an open field chamber (60 × 40 × 35 cm) under dim light. The mice were gently and individually introduced into the center of the chamber and were allowed to move around freely for 5 min. The movement parameters of the mice (mean speed of movement) were monitored and analyzed via a video camera connected to the Any-Maze animal tracking system software. The mean speed of movement of the mice was used to determine the locomotor activity of the mice following the surgery plus anesthesia.

### Brain tissue lysis and protein quantification

The brain tissues were harvested 12, 24, or 48 h after the surgery plus anesthesia. The cortex of each of the mice was homogenized on ice using an immunoprecipitation buffer (10 mM Tris-HCl, pH 7.4, 150 mM NaCl, 2 mM EDTA, 0.5% Nonidet P-40) plus protease inhibitors (1 μg/ml aprotinin, 1 μg/ml leupeptin, 1 μg/ml pepstatin A). The lysates were collected, centrifuged at 12,000 rpm for 15 min, and quantified for total protein by the bicinchoninic acid (BCA) protein assay kit (Pierce, Iselin, NJ). The brain tissues were then subjected to Western blot analysis as described by Xie et al. (2008).

### Western blot analysis

Total α-synuclein (T-α-synuclein) antibody (1:1000 dilution; Abcam, Cambridge, MA, Cat. Number: ab1903) was used to recognize T-α-synuclein (16 kDa). S100β antibody (1:1000 dilution; Abcam, Cambridge, MA, Cat. Number: ab52642) was used to recognize S100β (11 kDa). Antibody anti-β-Actin (1:10,000, Sigma, St. Louis, MO) was used to detect β-Actin (42 kDa). Western blot quantification was performed as described by Xie et al. (2008). One hundred percent of the protein level changes on the Y-axis of the figures refer to the control condition for the purpose of comparison with experimental conditions.

### Statistics

Data were expressed as means ± standard deviation (SD). The sample number was 12 per group for the behavioral tests and 6 per group for the Western blot studies. The power calculation was performed using the information we collected from a preliminary study, conducted under the same conditions. Based on the preliminary data, assuming a two-sided Student's *t*-test and a sample size of 6 and 12 for each control and treatment group for the Western blot, and attention and movement studies, respectively, a 90% power and a 95% significance level is obtained. Given that different groups of mice were used for the studies at 12, 24, and 48 h after the surgery plus anesthesia, a Student's *t*-test was used to assess the difference in the levels of attention, movement of the mice, and the T-α-synuclein and S100β levels in the brain tissues of the mice between the control (sham) condition and the treatment (surgery plus anesthesia) condition. The nature of the hypothesis testing was two-tailed. *P*-values less than 0.05 were considered statistically significant. SAS software (Cary, NC) and Prism 6 software (La Jolla, CA) were used to analyze the data.

## Results

### Surgery plus anesthesia decreased attention levels in the mice 24 H after the surgery plus anesthesia

**Figure 1:**
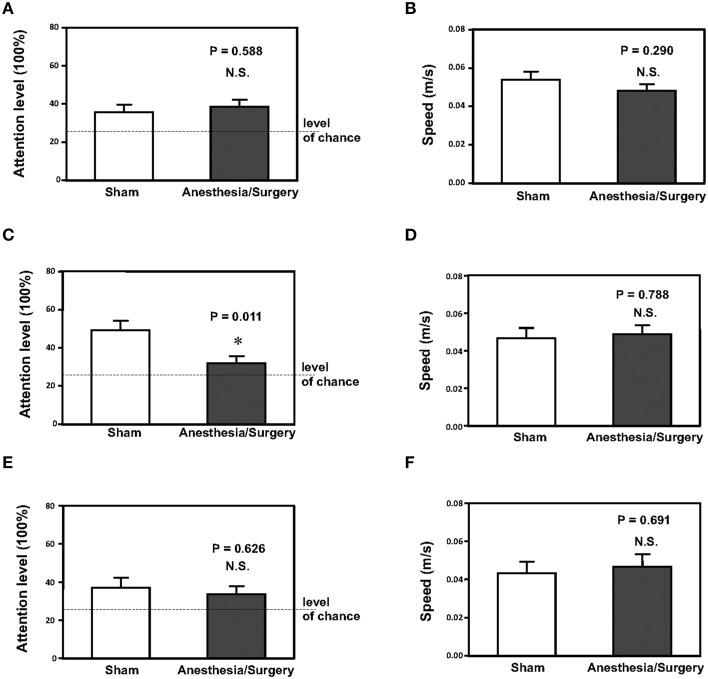
**Surgery plus anesthesia decreases the attention level at 24, but not 12 or 48 h after the surgery plus anesthesia in mice. (A)** Surgery plus anesthesia (black bar) does not decrease the attention level as compared to the sham condition (white bar) in mice 12 h after the surgery plus anesthesia. **(B)** Surgery plus anesthesia (black bar) does not decrease the speed of movement as compared to the sham condition (white bar) in mice 12 h after the surgery plus anesthesia. **(C)** Surgery plus anesthesia (black bar) decreases the attention level as compared to the sham condition (white bar) in mice 24 h after the surgery plus anesthesia. **(D)** Surgery plus anesthesia (black bar) does not decrease the speed of movement as compared to the sham condition (white bar) in mice 24 h after the surgery plus anesthesia. **(E)** Surgery plus anesthesia (black bar) does not decrease the attention level as compared to the sham condition (white bar) in mice 48 h after the surgery plus anesthesia. **(F)** Surgery plus anesthesia (black bar) does not decrease the speed of movement as compared to the sham condition (white bar) in mice 48 h after the surgery plus anesthesia. *N* = 12 in the control condition group and *N* = 12 in the surgery plus anesthesia group. ^*^P < 0.05.

We assessed whether surgery plus anesthesia could affect the behavioral changes including attention level in the mice. The mice had open abdominal surgery plus isoflurane anesthesia as described in the Materials and Methods Section. Then, we measured the effects of the surgery plus anesthesia on attention level 12 (Figures 1A,B), 24 (Figures 1C,D) and 48 (Figures 1E,F) h after the surgery plus anesthesia. As can be seen in Figure 1, the surgery plus anesthesia (black bar, Figure 1A) did not significantly alter the attention level of the mice compared to the sham condition (white bar, Figure 1A) 12 h after the surgery plus anesthesia (Figure 1A, *P* = 0.588, Student's *t*-test, *N* = 12 in each group). The surgery plus anesthesia did not significantly alter the speed of the mice's movements 12 h after the surgery plus anesthesia (Figure 1B, *P* = 0.290, Student's *t*-test, *N* = 12 in each group).

However, the surgery plus anesthesia (black bar, Figure 1C) decreased the level of attention compared to the sham condition (white bar, Figure 1C) in the mice 24 h after the surgery plus anesthesia: 49% ± 5 vs. 33% ± 2.9, *P* = 0.011 (Student's *t*-test, *N* = 12 in each group). The surgery plus anesthesia did not significantly alter the speed of movement (Figure 1D, *P* = 0.788, Student's *t*-test, *N* = 12 in each group) in the mice 24 h after the surgery plus anesthesia. These results showed that the surgery plus anesthesia did not impair the locomotor activity of the mice, and that the surgery plus anesthesia-induced loss of attention was not the result of impairment of the locomotor activity in the mice.

Finally, the surgery plus anesthesia altered neither the attention level in the mice nor the speed of the mice's movement, compared to the sham condition, 48 h after the surgery plus anesthesia (Figures 1E,F). Taken together, these results suggested that the surgery plus anesthesia might induce loss of attention for the mice in a time-dependent manner without impairment of locomotor activity.

### Surgery plus anesthesia increased the protein levels of total α-synuclein (T-α-synuclein) in the brain tissues of mice 12 h after the surgery plus anesthesia

**Figure 2:**
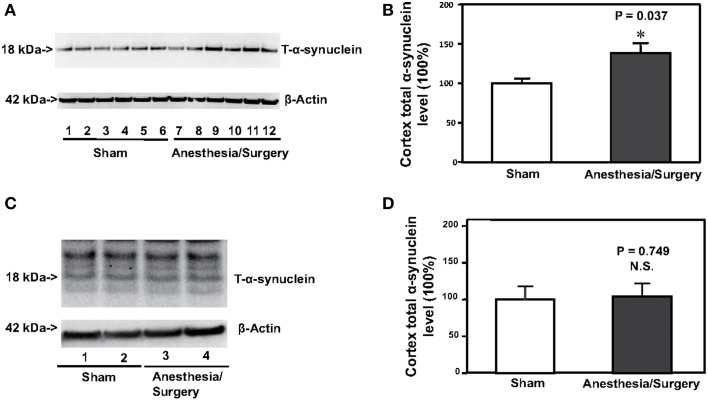
**Surgery plus anesthesia increases the levels of total α-synuclein (T-α-synuclein) in the cortex of mice 12 h after the surgery plus anesthesia. (A)** Western blot analysis shows that the surgery plus anesthesia (lanes 7–12) increases the levels of T-α-synuclein in the cortex of mice as compared to the sham condition (lanes 1–6) 12 h after the surgery plus anesthesia. There is no significant difference in the levels of β-Actin in the cortex of mice between the sham condition (lanes 1–6) and the surgery plus anesthesia (lanes 7–12). **(B)** Quantification of the Western blot shows that the surgery plus anesthesia (black bar) increases the levels of T-α-synuclein in the cortex of mice as compared to the control condition (white bar) 12 h after the surgery plus anesthesia. **(C)** Western blot analysis shows that the surgery plus anesthesia (lanes 3 and 4) does not increase the levels of T-α-synuclein in the cortex of mice as compared to the sham condition (lanes 1 and 2) 24 h after the surgery plus anesthesia. There is no significant difference in the levels of β-Actin in the cortex of mice between the sham condition (lanes 1 and 2) and the surgery plus anesthesia (lanes 3 and 4). **(D)** Quantification of the Western blot shows that the surgery plus anesthesia (black bar) does not increase the levels of T-α-synuclein in the cortex of mice as compared to the control condition (white bar) 24 h after the surgery plus anesthesia. *N* = 6 in the control condition group and *N* = 6 in the surgery plus anesthesia group. ^*^P < 0.05.

Increases in the levels of α-synuclein have been reported to be associated with postoperative delirium in humans (Sunwoo et al., 2013). Next, we determined the effects of the surgery plus anesthesia on the levels of T-α-synuclein in the cortex of mice. Immunoblotting of T-α-synuclein showed that the surgery plus anesthesia (lanes 7–12) induced a visible increase in the levels of bands in the Western blot representing T-α-synuclein compared to the sham condition (lanes 1–6) in the cortex of the mice (Figure 2A). There was not a significant difference in the β-Actin levels in the cortex of the mice between the surgery plus anesthesia condition and the sham condition. Quantification of the Western blot, based on the ratio of T-α-synuclein levels to β-Actin levels, showed that the surgery plus anesthesia increased the T-α-synuclein level in the cortex of mice compared to the sham condition: 100% ± 13.7 vs. 139% ± 33.5, *P* = 0.037 (Figure 2B, Student's *t*-test, *N* = 6 in each group). The surgery plus anesthesia did not increase the T-α-synuclein level in the cortex of mice, compared to the sham condition, 24 h after the surgery plus anesthesia (Figures 2C,D). These results suggested that the surgery plus anesthesia enhanced the T-α-synuclein levels in the brain tissue of the mice in a time-dependent manner.

### Surgery plus anesthesia increased the protein levels of S100β in the brain tissue of mice 12 H after the surgery plus anesthesia

**Figure 3:**
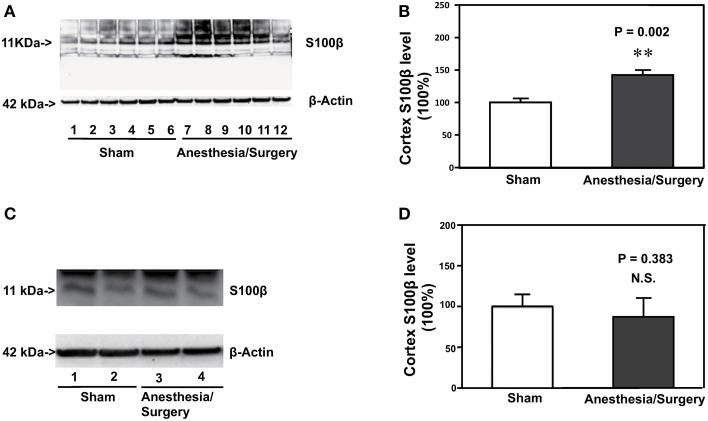
**Surgery plus anesthesia increases the levels of S100β in the cortex of mice 12 h after the surgery plus anesthesia. (A)** Western blot analysis shows that the surgery plus anesthesia (lanes 7 to 12) increases the levels of S100β in the cortex of mice as compared to the sham condition 12 h after the surgery plus anesthesia (lanes 1 to 6). There is no significant difference in the levels of β-Actin in the cortex of mice between the sham condition (lanes 1 to 6) and the surgery plus anesthesia 12 h after the surgery plus anesthesia (lanes 7 to 12). **(B)** Quantification of the Western blot shows that the surgery plus anesthesia (black bar) increases the levels of S100β in the cortex of mice as compared to the control condition 12 h after the surgery plus anesthesia (white bar). **(C)** Western blot analysis shows that the surgery plus anesthesia (lanes 3 and 4) does not increase the levels of S100β in the cortex of mice as compared to the sham condition 24 h after the surgery plus anesthesia (lanes 1 and 2). There is no significant difference in the levels of β-Actin in the cortex of mice between the sham condition (lanes 1 and 2) and the surgery plus anesthesia 24 h after the surgery plus anesthesia (lanes 3 and 4). **(D)** Quantification of the Western blot shows that the surgery plus anesthesia (black bar) does not increase the levels of S100β in the cortex of mice as compared to the control condition 24 h after the surgery plus anesthesia (white bar). *N* = 6 in the control condition group and *N* = 6 in the surgery plus anesthesia group. ^**^P < 0.01.

Increase in the S100β levels has been reported to be associated with postoperative delirium (Hall et al., 2013; Khan et al., 2013). Therefore, we assessed the effects of the surgery plus anesthesia on the S100β levels in the cortex of the mice. Immunoblotting of S100β showed that the surgery plus anesthesia (lanes 7–12) induced a visible increase in the levels of bands in the Western blot representing S100β as compared to the sham condition (lanes 1–6) in the cortex of the mice (Figure 3A). There was not a significant difference in the β-Actin levels in the cortex of the mice between the surgery plus anesthesia condition and the sham condition. Quantification of the Western blot, based on the ratio of S100β levels to β-Actin levels, showed that the surgery plus anesthesia increased the S100β level in the cortex of the mice as compared to the sham condition: 142% ± 7.7 vs. 100% ± 6, *P* = 0.002 (Figure 3B, Student's *t*-test, *N* = 6 in each group). The surgery plus anesthesia did not increase the S100β level in the cortex of the mice as compared to the sham condition 24 h after the surgery plus anesthesia (Figures 3C,D). These data suggested that the surgery plus anesthesia enhanced the S100β levels in the brain tissue of mice in a time-dependent manner.

## Discussion

In this system-establishment pilot study, we assessed whether surgery plus anesthesia was able to induce the behavioral changes and biochemical/cellular changes that are potentially associated with delirium. We found that the surgery plus anesthesia might induce behavioral changes including reduction in the attention levels in the mice 24, but not 12 or 48 h after the surgery plus anesthesia. Delirium symptoms include acute onset and fluctuating course, inattention, and either disorganized thinking or altered level of consciousness, or both of these last two symptoms. Therefore, the current results suggested that surgery plus anesthesia might induce certain aspects of delirium-like behavior (e.g., inattention) in the mice, pending further studies.

Millecamps et al. reported that inflammation and pain, both of which are associated with surgery, could induce loss of attention in rats (Millecamps et al., 2004). Culley et al. showed that systemic inflammation can also impair attention in rats (Culley et al., 2014). Consistently, we found that surgery plus anesthesia in the mice might induce loss of attention in the mice. These findings suggested that we might be able to use the established system of the surgery plus anesthesia in the mice to further set up animal models to study postoperative delirium. Other perioperative factors, including anesthesia (Culley et al., 2004a,b; Bianchi et al., 2008; Zhang et al., 2012), surgery plus anesthesia (Wan et al., 2007, 2010; Terrando et al., 2010), surgery under local anesthesia (Xu et al., 2014a,b), pain (Zhang et al., 2013; Yang et al., 2014), and also sleep deprivation (Zhu et al., 2012), have been shown to induce cognitive impairment in mice. Future studies might include assessments of whether or not these perioperative factors can also cause loss of attention in mice.

We were not able to detect loss of attention in the mice 12 or 48 h after the surgery plus anesthesia in the mice. These results might suggest that the surgery plus anesthesia-induced loss of attention observed in the mice may only occur at certain time(s) after the surgery plus anesthesia. However, because we only assessed the attention levels of the mice once per day, it is also possible that we missed the detection of loss of attention the first and third day after the surgery plus anesthesia. The mice could have had loss of attention at an earlier time (e.g., 6 h) after the surgery plus anesthesia. Future studies should thus implement more frequent time intervals, e.g., 6 and 9 h after the surgery plus anesthesia, in order to more accurately determine whether the surgery plus anesthesia induces loss of attention in mice.

The T-maze alternation task has been reported to assess working memory deficits in mice, and could therefore be used to assess delirium-like behavior in mice following systemic inflammation (Field et al., 2012; Murray et al., 2012; Griffin et al., 2013). This is because the nature of the working memory deficits is acute and transient, and causes impairments in attention, recall, and short-term/working memory, which may include the key features of delirium. However, the T-maze alternation task requires the training of mice (e.g., 10 trials of training) to be included in the study before working memory can be appropriately tested. Similarly, the AST is also limited by the fact that it necessitates training of the rodents before delirium-like behavior can be detected (Culley et al., 2014).

The ideal behavioral test for determining loss of attention and other delirium-like behavior in rodents should (1) not require much training of the rodents and (2) be able to detect the more natural, subtle behavior of the animals. Future studies should include experiments to determine whether surgery plus anesthesia, as well as other perioperative factors, can alter other natural behaviors of mice that are associated with delirium. Results from the current pilot studies, which indicate that surgery plus anesthesia may trigger changes in certain domains of delirium-like behavior (e.g., loss of attention), might help to facilitate the final establishment of postoperative delirium animal model(s).

Elevated levels of α-synuclein could potentially be associated with postoperative delirium in humans (Sunwoo et al., 2013). S100β has also been reported to be potentially associated with delirium in humans (Hall et al., 2013; Khan et al., 2013). We found that the surgery plus anesthesia increased the levels of T-α-synuclein and S100β in the cortex of mice, as well as induced loss of attention in the mice. These data suggest that the surgery plus anesthesia in the mice might induce both behavioral and biochemical/cellular changes associated with delirium. Future experiments should attempt to further test the hypothesis that changes in the levels of α-synuclein and S100β might be, at least partially, the underlying mechanisms of postoperative delirium. We exclusively assessed the effects of surgery plus anesthesia on the levels of α-synuclein and S100β in mouse cortex because interactions between the prefrontal and the parietal cortex have been associated with attention (Corbetta and Shulman, 2002; Sarter and Paolone, 2011).

Interestingly, the surgery plus anesthesia-induced elevation in the levels of T-α-synuclein and S100β occurred at 12, but not 24 or 48 h after the surgery plus anesthesia, whereas the surgery plus anesthesia-induced loss of attention only occurred at 24, but not 12 or 48 h after the surgery plus anesthesia in the mice. The reasoning behind this dissociation between the biochemical/cellular changes and the behavioral changes is not known at the present time. We have postulated that the surgery plus anesthesia-induced elevation in the levels of T-α-synuclein and S100β may cause other time-dependent changes, e.g., synaptic loss or dysfunction, leading to loss of attention. Future research should include time-course studies (3, 6, 9, 12, 24, and 48 h after surgery plus anesthesia) to determine the changes in T-α-synuclein, S100β, synapses and behavior in mice, in order to test this hypothesis. Moreover, inhibition of cholinergic neurotransmission has been suggested to contribute to delirium, including loss of attention (Tune et al., 1981; Trzepacz, 1996, 2000; Field et al., 2012). Therefore, the future studies should also include experiments to determine whether the surgery plus anesthesia can induce loss of attention in mice by inhibiting cholinergic neurotransmission.

Postoperative delirium occurs in patients of all ages. We therefore used a wide age range of mice (2- to 8-months-old) in the current studies to establish a system to detect attention deficit, one of the symptoms of delirium. Mice of different ages may have different changes in cognitive function following the surgery plus anesthesia (Xu et al., 2014a,b). In future studies, we will use the established system to determine whether mice with different ages have different changes in attention levels following the surgery plus anesthesia.

Different anesthetics may have different effects on cognitive function and attention levels. The observed effect in the current study might be isoflurane specific. In the future studies, we will compare the effects of surgery under different anesthetics on the attention in mice. Surgery itself could induce neuroinflammation, accumulation of Aβ and cognitive impairment (Xu et al., 2014a,b), thus, it is likely that surgery also contributes to the observed changes in attention levels in the mice. Future studies should include the assessments of the effects of anesthesia or surgery alone on the attention level of mice.

Our study has several limitations. First, we did not determine the attention levels of mice at many time points, e.g., 6 h after the surgery plus anesthesia. However, the primary objective of the current pilot study was to establish a system indicating that surgery plus anesthesia was able to decrease the attention levels in the mice. The data obtained from these studies would then lead to systematic investigations of the effects of surgery plus anesthesia, as well as other perioperative factors (e.g., pain), on attention level in mice. Second, we only assessed the effects of surgery plus anesthesia on the levels of α-synuclein and S100β in the cortex of mice. Surgery plus anesthesia could have different effects on the levels of α-synuclein and S100β in different regions of the brain, e.g., hippocampus, as well. Future investigations should look into the potential effects of surgery plus anesthesia, as well as other perioperative factors, on the levels of α-synuclein and S100β in other regions of the brain, utilizing our established system. Third, surgery plus anesthesia may have different impacts on the postoperative behavioral changes, including delirium. The current studies only determined the combined effects of surgery plus anesthesia on loss of attention in mice. Future studies will include assessments of the effects of anesthesia or surgery alone on the attention level of mice. Finally, the reduction in the new object exploration time may represent both loss of attention and impairment in learning and memory in mice. However, there are not behavioral tests that can exclusively assess loss of attention in mice.

In conclusion, our current pilot studies assessed the effects of surgery plus anesthesia on the behavioral changes including attention level in the mice, as well as on the levels of α-synuclein and S100β in the brain tissues of these mice. The results of these studies suggested that surgery plus anesthesia could induce behavioral changes (e.g., loss of attention) as well as biochemical/cellular changes (e.g., increase in the levels of α-synuclein and S100β) in mice, which have been reported to be associated with delirium. These data suggested that we could use the anesthesia and surgery in the mice as a system to develop an animal model to study postoperative delirium in rodents in the long run, which would likely lead to further investigations ultimately aimed at establishing an animal model(s) that illustrates the neuropathogenesis of postoperative delirium.

## Author contributions

ZX, YD, YZ, MC, NY, and EM. conceived and designed the project. QR and MP performed all the experiments and prepared the figures. ZX and YZ wrote the manuscript. All authors reviewed the manuscript.

### Conflict of interest statement

The authors declare that the research was conducted in the absence of any commercial or financial relationships that could be construed as a potential conflict of interest.
